# Outcome of resveratrol and resveratrol with donepezil combination on the β-amyloid plaques and neurofibrillary tangles in Alzheimer’s disease

**DOI:** 10.1007/s13205-024-04034-2

**Published:** 2024-08-01

**Authors:** Y. Lakshmisha Rao, B. Ganaraja, Pooja K. Suresh, Teresa Joy, Sheetal D. Ullal, Poornima A. Manjrekar, B. V. Murlimanju, B. Gaurav Sharma, Amit Massand, Amit Agrawal

**Affiliations:** 1 Department of Anatomy, Kasturba Medical College, Mangalore, Manipal Academy of Higher Education, Manipal, Karnataka India; 2 Department of Physiology, Kasturba Medical College, Mangalore, Manipal Academy of Higher Education, Manipal, Karnataka India; 3 Department of Pathology, Kasturba Medical College, Mangalore, Manipal Academy of Higher Education, Manipal, Karnataka India; 4 Department of Anatomy, American University of Antigua College of Medicine, Jabberwock Beach Road, University Park, Coolidge, Antigua Antigua and Barbuda; 5 Department of Pharmacology, Kasturba Medical College, Mangalore, Manipal Academy of Higher Education, Manipal, Karnataka India; 6 Department of Biochemistry, Kasturba Medical College, Mangalore, Manipal Academy of Higher Education, Manipal, Karnataka India; 7 Senior Registrar in Trauma and Orthopaedic Surgery, Hampshire Hospitals NHS Foundation Trust, Basingstoke and North Hampshire Hospital, Aldermaston Road, Basingstoke, RG24 9NA UK; 8 Department of Anatomy, Smt. B.K. Shah Medical Institute and Research Centre, Sumandeep Vidyapeeth, Piparia, Vadodara, Gujarat India; 9 Department of Neurosurgery, All India Institute of Medical Sciences, Saket Nagar, Bhopal, Madhya Pradesh India

**Keywords:** Alzheimer’s disease, β-amyloid, Hippocampus, Neurofibrillary tangles, Resveratrol

## Abstract

The goal of this research was to study the effect of different doses of resveratrol (RS) and RS with donepezil (DPZ) on the deposition of amyloid beta (Aβ) and neurofibrillary tangles (NFTs) in colchicine-induced Alzheimer’s disease (AD) brain. The study included three months old male Albino *Wistar* rats and consisted of six animal groups: AD model (group 1), treatment groups, RS 10 mg/kg body weight (group 2), RS 20 mg/kg body weight (group 3), RS 10 mg/kg body weight along with DPZ 1 mg/kg body weight (group 6), prophylaxis groups, RS 10 mg/kg body weight (group 4) and RS 20 mg/kg body weight (group 5). In the treatment groups, RS was given for 7 consecutive days from the day of induction of AD, and in the prophylaxis groups, we started RS 7 days even before the induction of AD and continued for seven days after the induction. The number of Aβs and NFTs at the frontal region, cornu ammonis (CA) 1,2,3,4 and dentate gyrus regions of hippocampus were evaluated. The immunohistochemical analysis was performed by using mouse anti-β-amyloid antibody for the Aβ plaques and polyclonal rabbit anti-human tau for the tau-positive neurons. The present study observed the accumulation of Aβ plaques and tau-positive neurons in the AD model. However, their numbers were significantly decreased in the treatment groups (*p* < 0.001). The best results were observed when RS 10 mg was given prophylactically (*p* < 0.01) and RS along with DPZ (*p* < 0.001), suggesting the neuroprotective effect of RS and its synergistic effect with the DPZ.

## Introduction

The hippocampus is a part of the limbic system, which is essential for learning and memory. Its sub-regions, dentate gyrus, and cornu ammonis (CA), help in episodic memory (Rao et al. 2022). The oxidative stress leading to intracellular accumulation of hyperphosphorylated tau protein as neurofibrillary tangle (NFT) and extracellular deposition of beta-amyloid (Aβ) plaque at the hippocampus and other parts of the brain are the pathological features of Alzheimer’s disease (AD) (Rao et al. 2020; Huang et al. 2016; Chu 2012). Usually, the tau is highly soluble and unfolded proteins found in the axon and dendrites of neurons, which maintain the neuronal structure and transport when associated with the microtubules (Pradeepkiran and Reddy 2019; Mietelska-Porowska et al. 2014). Tau protein helps in the stabilization of the microtubules; however, it becomes toxic in its phosphorylated form (Ballatore et al. 2007). So, it is evident that the function of the tau protein is dependent on its state of phosphorylation. Hyperphosphorylated tau decreases the stability of microtubules resulting in their disruption and aggregation of oligomers, paired helical filaments (Yu et al. 2018). The basic pathophysiology of AD is neuronal inflammation due to oxidative stress and the formation of Aβ and tau. However, the cause of AD remains idiopathic due to the involvement of multiple factors (Reddy et al. 2017; Reddy et al. 2012).

**Fig. 1:**
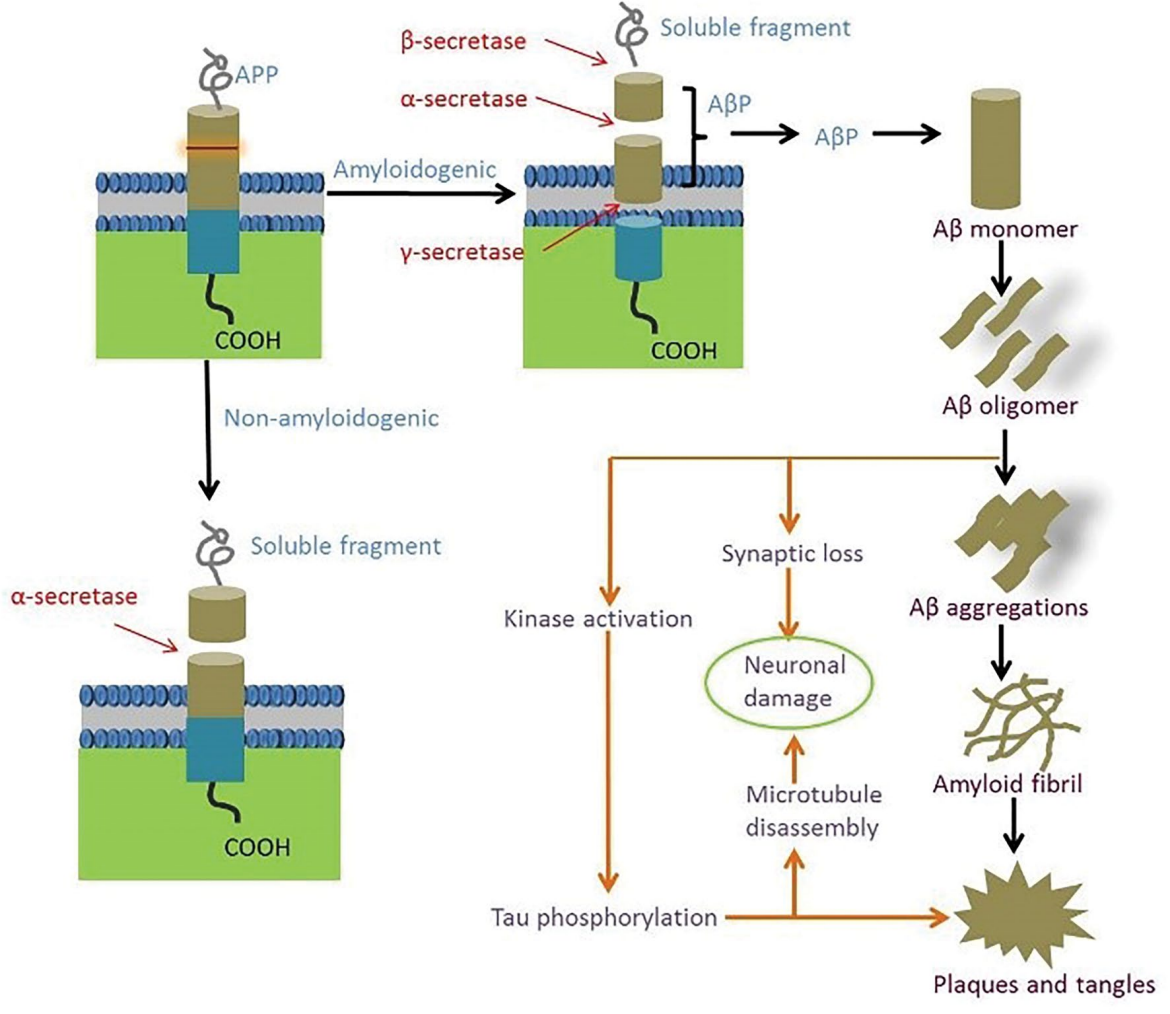
Schematic representation of formation of amyloid beta plaque and neurofibrillary tangles due to APP cleavage by the β and γ secretases at abnormal sites

Amyloid is a protein fragment that is normally produced by the body. Aβ is a peptide of 36 to 43 amino acids. It is snipped from an amyloid precursor protein (APP). The healthy brain will eliminate these protein fragments. In AD, the fragments are insoluble and they will form plaques. Abnormal cleavage of APP will lead to amyloid plaques, which are insoluble peptide clumps. Usually, the three enzymes named γ secretase, β secretase, and α secretase will cleave the APP. In AD brain, a variant γ secretase cleaves the APP at an incorrect place, producing 42 amino acid peptides named, Aβ 42 or Aβ. This Aβ is a non-soluble aggregate, and forms β amyloid plaques (Morrison and Lyketsos 2005). This abnormal deposition of Aβ plaques in the brain leading to neurodegeneration is termed as the ‘amyloid cascade hypothesis’ (Fig. 1). Hardy and Higgins (1992) proposed this amyloid cascade hypothesis in AD pathology for the first time. The amyloid hypothesis attributes to the neurodegeneration and cognitive impairment (Ran et al. 2020; Selkoe and Hardy 2016; Herrup 2015; Karran et al. 2011). Accumulation of Aβ plaques will lead to the formation of NFTs, vascular damage, and cellular death, eventually resulting in dementia. This variant cleavage site by γ secretase results in forming of Aβ peptides with different lengths like Aβ_40_ and Aβ_42_. These forms of Aβ are commonly seen in human brains. Hence, APP and PSEN 1/2 mutations are responsible for familial AD (Ricciarelli and Fedele 2017; Hardy and Higgins 1992).

Normal tau is an unfolded and highly soluble protein accumulated in axons and dendrites of neurons (Mietelska-Porowska et al. 2014). These tau proteins when associated with microtubules, help maintain the neuronal structure and neuronal transport (Pradeepkiran and Reddy 2019). Though tau protein helps stabilize the microtubules, its phosphorylated form becomes cytotoxic (Ballatore et al. 2007). Hence the function of tau protein is dependent on its phosphorylation state. Tau conformation and balance between the phosphate and kinase will determine the binding of the phosphate group into the tau protein. Altered tau conformation will lead to hyper-phosphorylation. In addition, the binding capacity of tau proteins into the microtubules is reduced. These two phenomena play a crucial role in the process of neuro-degeneration (Mietelska-Porowska et al. 2014). The hyper activated phosphatases will lead to paired helical filament and NFT formation (Pradeepkiran and Reddy 2019).

Neurodegenerative diseases like AD are associated with chronic inflammation of the brain, which involves progressive structural and functional neuronal loss at the subcellular level (Danışman et al. 2023). Neurons require a high volume of energy, which relies on the function of mitochondria. If the mitochondria become non-functional, it may lead to necrosis and apoptosis (Rai et al. 2020). The damage to mitochondrial DNA can cause deprivation of ATP, oxidative stress, production of reactive oxygen species (ROS) and cell death. The proinflammatory factors like TNF-α, IL-1β, and IL-6 are thought to play significant role in the neuroinflammation (Rai et al. 2018). Thus regulating the inflammatory response along with the growth and differentiation of neurons is crucial in preventing dementia. There is no permanent cure for AD. However, it may be preventable. The modified lifestyle including the good physical activity and diet can help dementia. It was reported that, mild to moderate consumption of red wine helped the dementia patients (Lange 2018), as grapes and wine were neuroprotective, and they decreased the decline in cognition and aging.

**Fig. 2:**
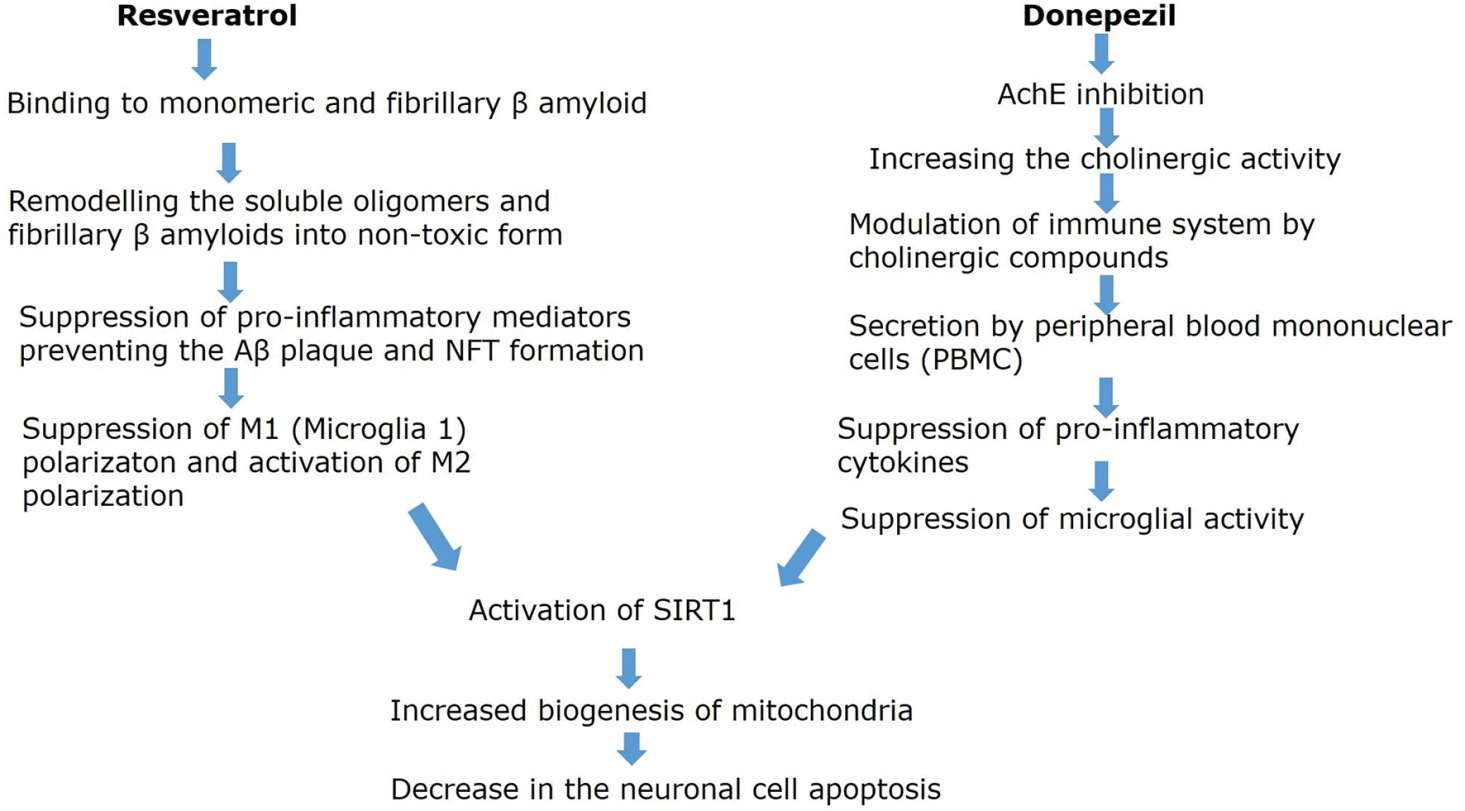
Possible cytomolecular mechanism of action of combined regime of resveratrol with donepezil in Alzheimer’s diseases

The polyphenols found in grapes were administered as a prophylactic treatment for dementia. Resveratrol (RS) is a polyphenol which is found abundantly in plant sources like grapes, groundnuts, jack fruit, wine, and berries. It is also abundant in green tea, pomegranates, rhubarbs, and other herbs. It was reported that, the grape fruit extract augmented with RS has prevented the herbicide induced hippocampal damage (Dasgupta and Bandyopadhyay 2013). RS is considered as an antioxidant, anti-inflammatory and a potential neuroprotective. These are the rationale for choosing the RS as one of the treatment drugs in this study. Donepezil (DPZ) is one of the initial drugs approved by the United States, Food and Drug Administration (FDA). It has a benzyl piperidine nucleus, which is oriented towards the catalytic active site of AChE (Tripathi et al. 2019a). AChE is responsible for the hydrolysis of ACh in neuronal synapses and is one of the primary targets for the treatment of dementia (Srivastava et al. 2019). DPZ is already in clinical use for the management of dementia and AD (Lee et al. 2019). Yet, the effectiveness of DPZ in AD could be more satisfactory. Due to multifactorial pathogenesis, the trend of current drug therapy is to design multifunctional inhibitors for the disease progression, which can interact with more than one target together (Shrivastava et al. 2022). Several studies observed that administration of two different drugs gives better results than a single drug in multifactorial disorders, especially when the two drugs have different mechanisms of action. For example, Sato et al. (2019) observed better success with the use of two different medications in relieving the cognitive dysfunction and psychosis. The literature review suggested that there are not enough studies available with the administration of two different drugs for an AD model, and the synergistic effect of their combination was not being studied. Our study administered different doses of RS and RS along with DPZ, and their role in ameliorating the inflammation is evaluated. The combined treatment of RS and DPZ for the AD and evaluation of Aβ and NFTs is novel in the scientific literature. This study was undertaken as the reports are not available when these two drugs are administered together, which is the originality of this study and necessitates this research. The possible mechanism of actions of combined regime of RS with DPZ on neuroimmune mechanisms in AD, with cytomolecular control of neuro-inflammation pathophysiology is represented in Fig. 2.

Colchicine-induced neurodegeneration is a well-established animal model of AD. It increases the expression of intraneuronal expression of Aβ and tau in the medial prefrontal cortex and hippocampus (Joy et al. 2019, 2018). The idealistic disease animal model should reiterate the symptoms and lesions, similar to the actual disease. Colchicine is basically an anti-inflammatory drug, which is cytotoxic as it binds to the tubulin molecules and disrupts the polymerization of microtubules. It prevents axoplasmic transport and causes neuronal loss by damaging the granule cells. This is ideal for the AD animal model in rats as it induces chronic neuroinflammation, memory and cognitive impairment, and development of tau and Aβ pathology after the intraventricular administration (Nazem et al. 2015). Hence, colchicine was considered to induce the AD model in this present research. The aim of this study was to evaluate the effect of different doses of RS and a combination of RS with DPZ on the formation of Aβ plaques and NFTs in the form of tau-positive neurons in the hippocampus and frontal cortex of the AD model. We hypothesize that the treatment gap that exists in AD therapy may be closed to some extent by adding RS to DPZ.

## Materials and methods

### Experimental design

The present study is a randomized case control study, which included animal models of albino *Wistar* rats. Each group of animals underwent stereotaxis surgery to inject colchicine intraventricularly at the brain to induce the AD. The biochemical analysis of lipid peroxidation did the confirmation of AD through estimation of malondialdehyde level and antioxidant activity through superoxide dismutase level (Rao et al. 2021). The treatment groups received the drugs immediate next day of the surgery till the seventh day. The prophylactic groups received the drugs starting from 7 days before the surgery and continued for 7 days after the surgery. The next day of the completion of treatment, the animals were sacrificed, and brain tissue was removed for the immunohistochemical analysis. The β-amyloid plaques and tau-positive neurons were counted, and the numerical variables were statistically analyzed.

### Inclusion and exclusion criteria

The male albino *Wistar* rats, which were 3 months of age and weighing approximately 220 to 250 gms at the begining of this research were included. The female rats and those with congenital anomalies were excluded.

### Animals

The rats were bred-in-house at our institutional animal house. A polypropylene cage was used to house the rats, and bedding was made with the paddy husk. The circadian rhythm for the animals was maintained as the rat room had 12 h of light and darkness each, alternatively (Shrivastava et al. 2018). The room temperature was maintained at 22 ± 3 °C, and the humidity was approximately 50 ± 10%. This controlled room environment enabled the maintenance of pathogen-free rats. Throughout the experiment, the rats were fed with water and food ad libitum. Before the commencement of this study, approval was taken from the institutional animal ethics committee, Kasturba Medical College, Mangalore (KMC/MNG/IAEC/22-2018). The study was performed per the national government guidelines of India for the utilization of animal models in the laboratory.

The study included the following animal groups, and each group had six rats (*n* = 6):

- **Group 1:** Colchicine-induced AD model (AD).
- **Group 2:** RS (10 mg/kg body wt), for 7 days after the induction of AD (RS 10).
- **Group 3:** RS (20 mg/kg body wt), for 7 days after the induction of AD (RS 20).
- **Group 4:** RS (10 mg/kg body wt), started 7 days before the induction of AD and continued till 7 days after the induction of AD (RS 10/10).
- **Group 5:** RS (20 mg/kg body wt), started 7 days before the induction of AD and continued till seven days after the induction of AD (RS 20/20).
- **Group 6:** RS (10 mg/kg body wt) and DPZ (1 mg/kg body wt) for 7 days after the induction of AD (DPZ + RS).

### Chemicals and drug administration

Colchicine was obtained from Sigma Alderich, Bengaluru (catalog number 9754), and RS was in pale yellow form (99.9% pure, product No. R0071), which was procured from Tokyo Chemical Industry Co. Ltd., Japan. Polyclonal rabbit anti-human tau was purchased from Dako Denmerk, Produktionsverg, Denmark. Mouse monoclonal anti Aβ protein was purchased from Bioscience Diagnostics. The rest of the reagents and chemicals utilized in this study are Sigma Aldrich, Saint Louis, MO, The United States of America.

The dosages of RS considered in this study were as per the previous research performed by earlier authors (Rao et al. 2021; Wiciński et al. 2020; Sharma and Gupta 2002). The dosage of DPZ is as per the previous neurological studies performed in the rat model, which observed the beneficial effect on cognitive function (Bansal et al. 2017; Shin et al. 2017). The normal saline was used as a solvent for the colchicine, RS, and DPZ. The stereotactic surgery was done to insert the colchicine into the lateral ventricle. Colchicine, 15 µg in a volume of 5 µl was injected through the Hamilton micro syringe, which was placed in Harvard apparatus infusion pump (Rao et al. 2023). The intraperitoneal route was preferred for the RS and the oral route for the DPZ. Groups 2 and 3 received the RS from the next day of induction of AD and continued till day 7. In groups 4 and 5, RS was started 1 week before the induction of AD and continued till day 7 after the induction. In group 6, RS was administered with DPZ for 7 days after the induction of the AD model.

### Tissue processing

Twenty-four hours after the end of treatment (day 8), the animals of all the groups were sacrificed by cervical decapitation. This is followed by the brain removal and immersion of the brain in 10% formalin for 48 h. The dehydration of the brain tissue was done by immersing in an ascending order of concentrations of methyl alcohol. To procure the frontal cortex, brain was cut coronally at the frontal lobe, and for the hippocampal region, the brain tissue was cut coronally, just behind the temporal pole. Paraffin blocks were prepared using an embedding bath, and microtomy was done to procure the sections of 6–7-µm thickness. Mounting was done on air-dried slides coated with poly-L-lysine.

### Staining method

**Fig. 3:**
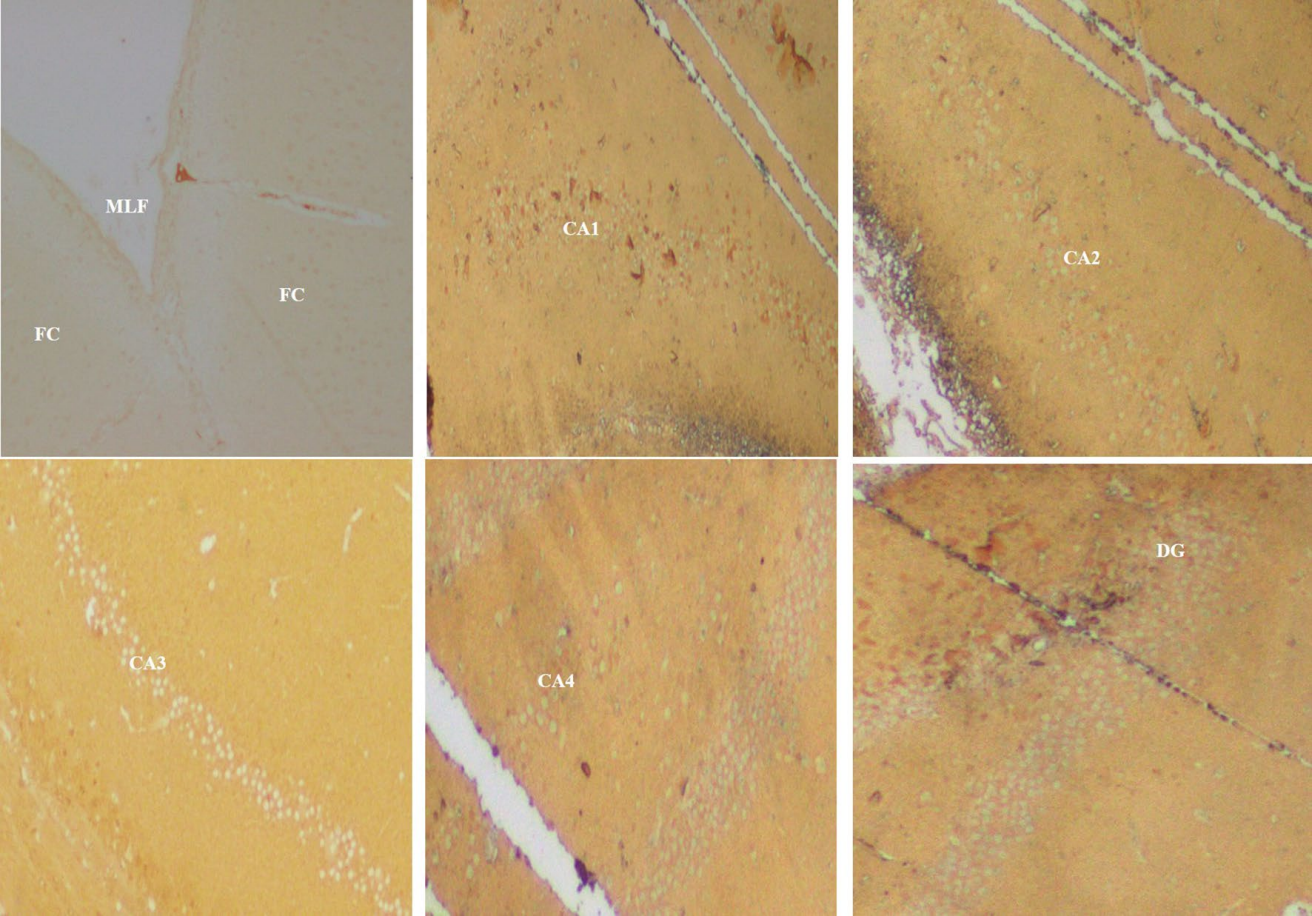
4 × view of the mouse monoclonal anti Aβ antibody immunohistochemical staining of the FC, CA1, CA2, CA3, CA4, and DG regions (FC-frontal cortex, MLF-median longitudinal fissure, CA-cornu ammonis, DG-dentate gyrus)

**Fig. 4:**
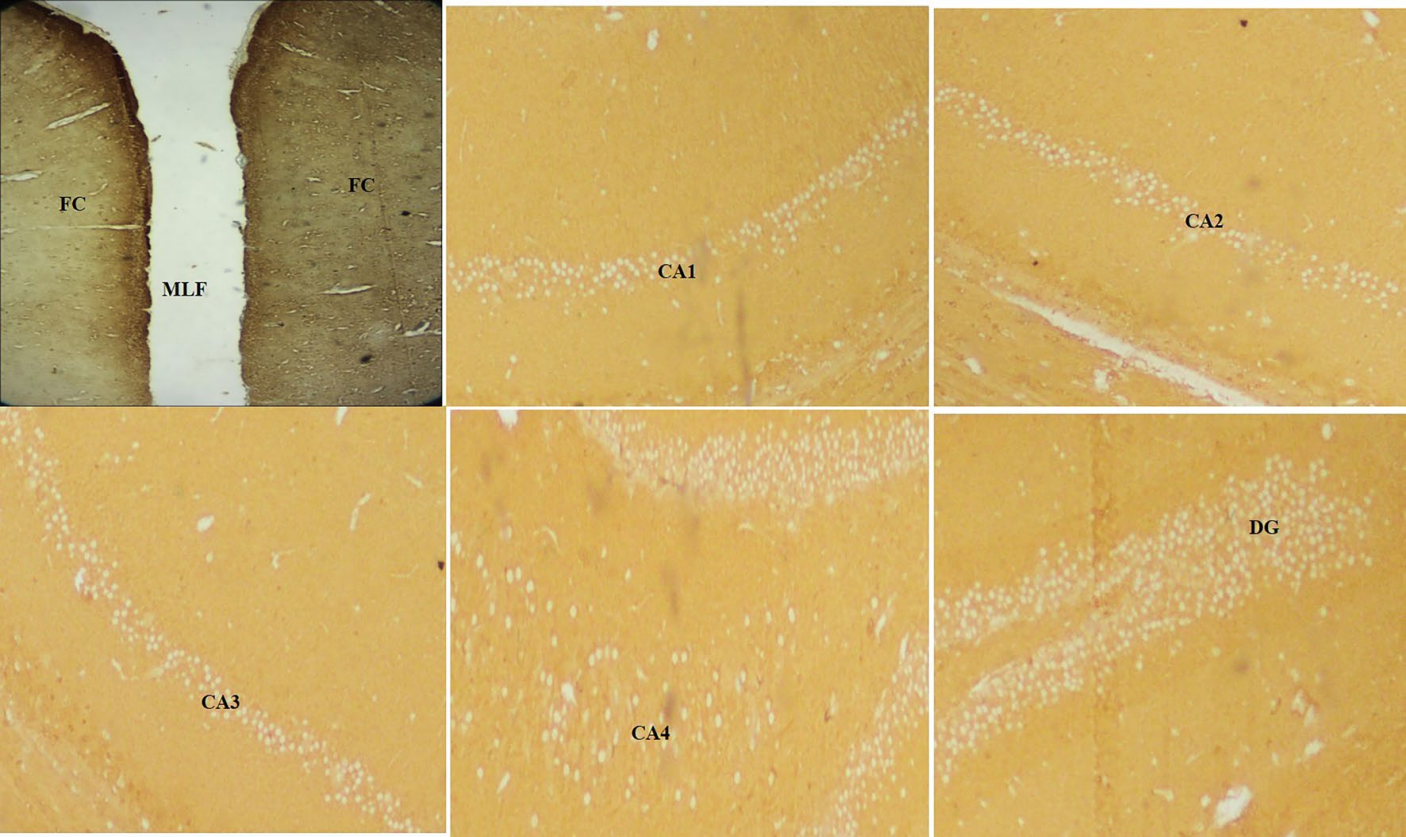
4 × view of the polyclonal rabbit anti-tau antibody immunohistochemical staining of the FC, CA1, CA2, CA3, CA4, and DG regions (FC-frontal cortex, MLF-median longitudinal fissure, CA-cornu ammonis, DG-dentate gyrus)

**Fig. 5:**
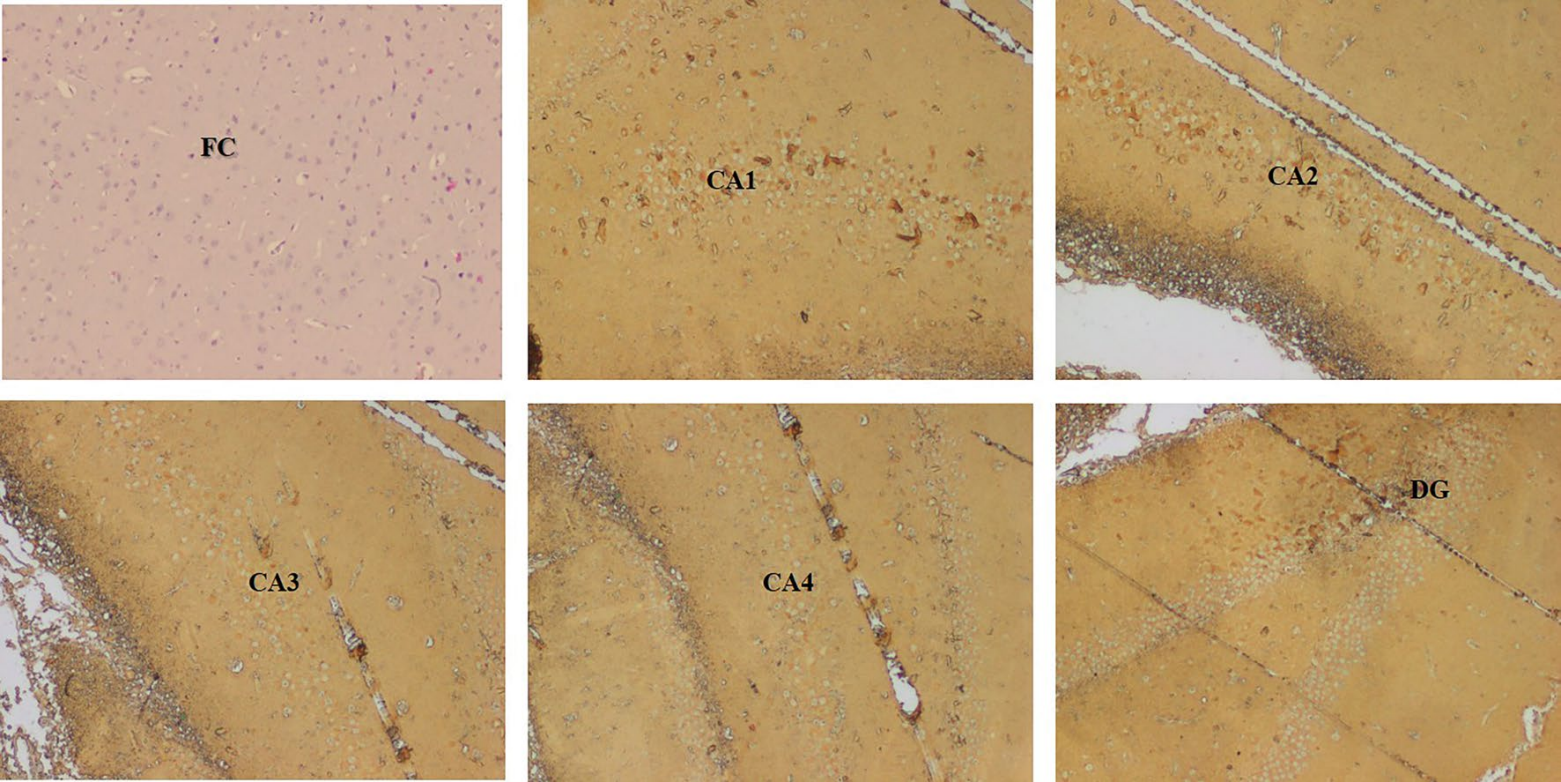
10 × view of the mouse monoclonal anti Aβ antibody immunohistochemical staining of the FC, CA1, CA2, CA3, CA4, and DG regions (FC-frontal cortex, CA-cornu ammonis, DG-dentate gyrus)

**Fig. 6:**
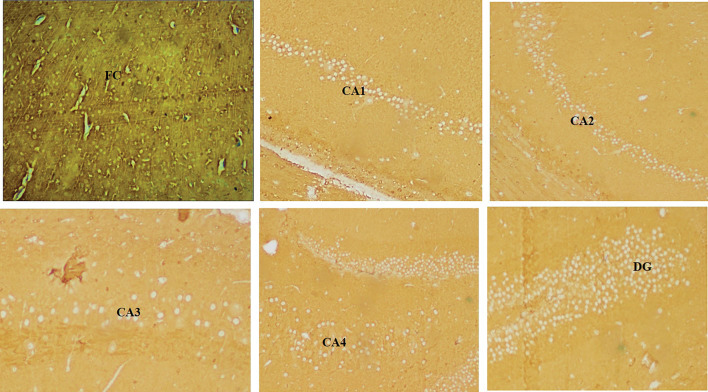
10 × view of the polyclonal rabbit anti-tau antibody immunohistochemical staining of the FC, CA1, CA2, CA3, CA4 and DG regions (FC-frontal cortex, CA-cornu ammonis, DG-dentate gyrus)

The immunohistochemical staining was performed by using the primary and second antibodies. The primary antibodies were mouse monoclonal anti-β-amyloid antibody (Fig. 3) for the Aβ plaques (Braak et al. 2011; Park et al. 2011) and polyclonal rabbit anti-human tau antibody (Fig. 4) for the tau-positive neurons (Gregor and Hotamisligil 2011). Overnight incubation was done at 4 °C, which was followed by the addition of a secondary antibody, goat-anti-rabbit IgG, and incubated again for an hour at room temperature. The 10 × view of the immunohistochemical staining of Aβ plaques and tau-positive neurons are shown in Figs. 5, 6, respectively.

### Scoring and estimation of Aβ plaque and NFT formation (tau-positive neurons)

The frontal cortex was identified as an area of the brain lying just lateral to the median longitudinal fissure. A 250 µ^2^ field was considered for the quantification of tau-positive neurons and Aβ at the frontal region of the cerebrum. The hippocampal formation was identified as per the description by Lorente de Nó (1934). The CA1 was nearer the entorhinal cortex, CA3 continued till the hilus fasciae dentatae, and CA2 was a narrow region between CA1 and CA3. The CA4 is the region between the arms of the fascia dentate. The dentate gyrus was identified by its ‘V’ shaped morphology (Amaral et al. 2007). In hippocampus regions (CA1, CA2, CA3, CA4), a 250 µm length area was chosen for the quantifications, and 150 µ^2^ was selected for the dentate gyrus (DG). In each image, immune-stained Aβ plaques were counted using NIS Elements Br version 4.30 software.

The Aβ plaques and tau-positive neurons were identified, and the counting was done according to the earlier reports (Joy et al. 2019, 2018; Shi et al. 2018). The observations were done at 20×magnification, and images of all the groups were captured by using a Nikon trinocular microscope (H600L). The NIS Elements (imaging software, Br version 4.30) was used for counting the Aβ plaques and tau-positive neurons (Madhyastha et al. 2002).

### Statistical analysis

The comparison of different groups of this study for the statistical significance was performed with the ‘one-way ANOVA test’ and post-hoc test, Bonferroni method by using the statistical software ‘EZR’. The statistical significance is categorized as highly significant for the ‘*p*’ values less than 0.001, moderately significance for the ‘*p*’ values less than 0.01, and significant if it is less than 0.05.

## Results

**Fig. 7:**
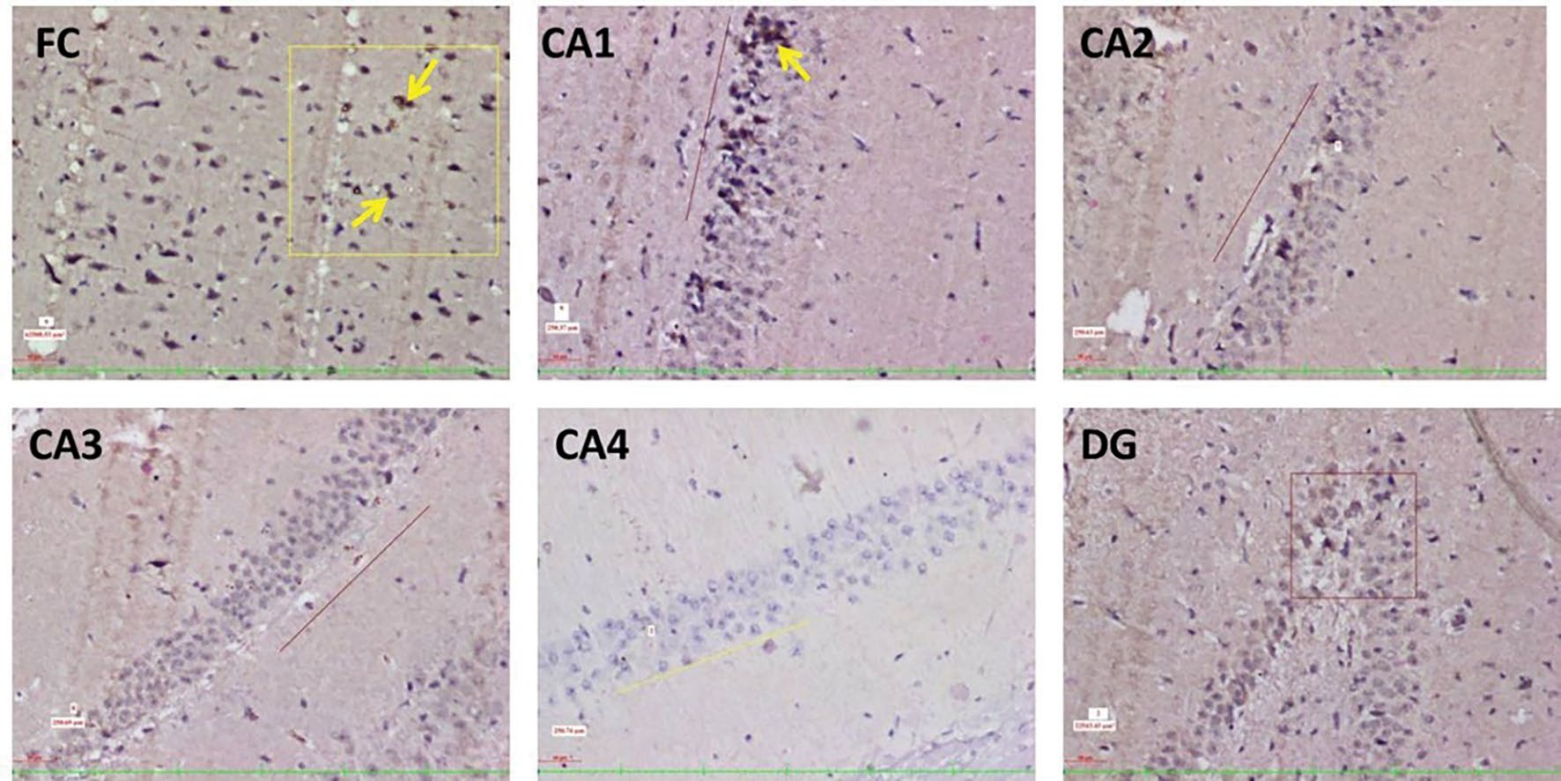
20 × view of the Aβ plaques observed at the FC, CA1, CA2, CA3, CA4, and DG regions (FC-frontal cortex; CA-cornu ammonis, DG-dentate gyrus, mouse monoclonal anti Aβ antibody immunohistochemical staining, lines dictate the length of cornu ammonis region selected for the counting; squares are the area of the frontal cortex and dentage gyrus selected for counting; the Aβ plaques are shown as arrows)

**Fig. 8:**
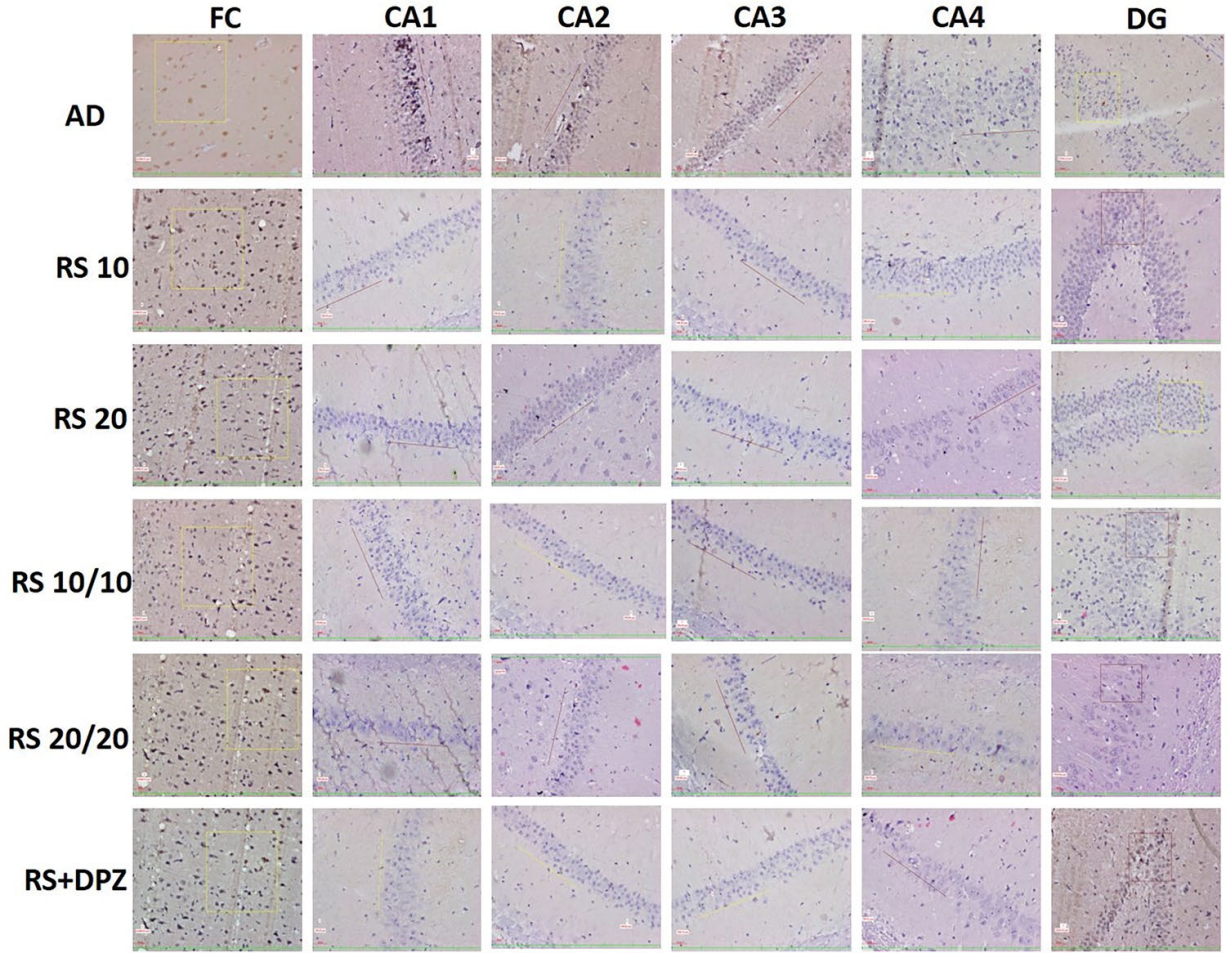
20 × view of the mouse monoclonal anti Aβ antibody immunohistochemical staining in different groups of this study showing the Aβ plaques observed at the FC, CA1, CA2, CA3, CA4 and DG regions (FC-frontal cortex; CA-cornu ammonis; DG-dentate gyrus)

**Fig. 9:**
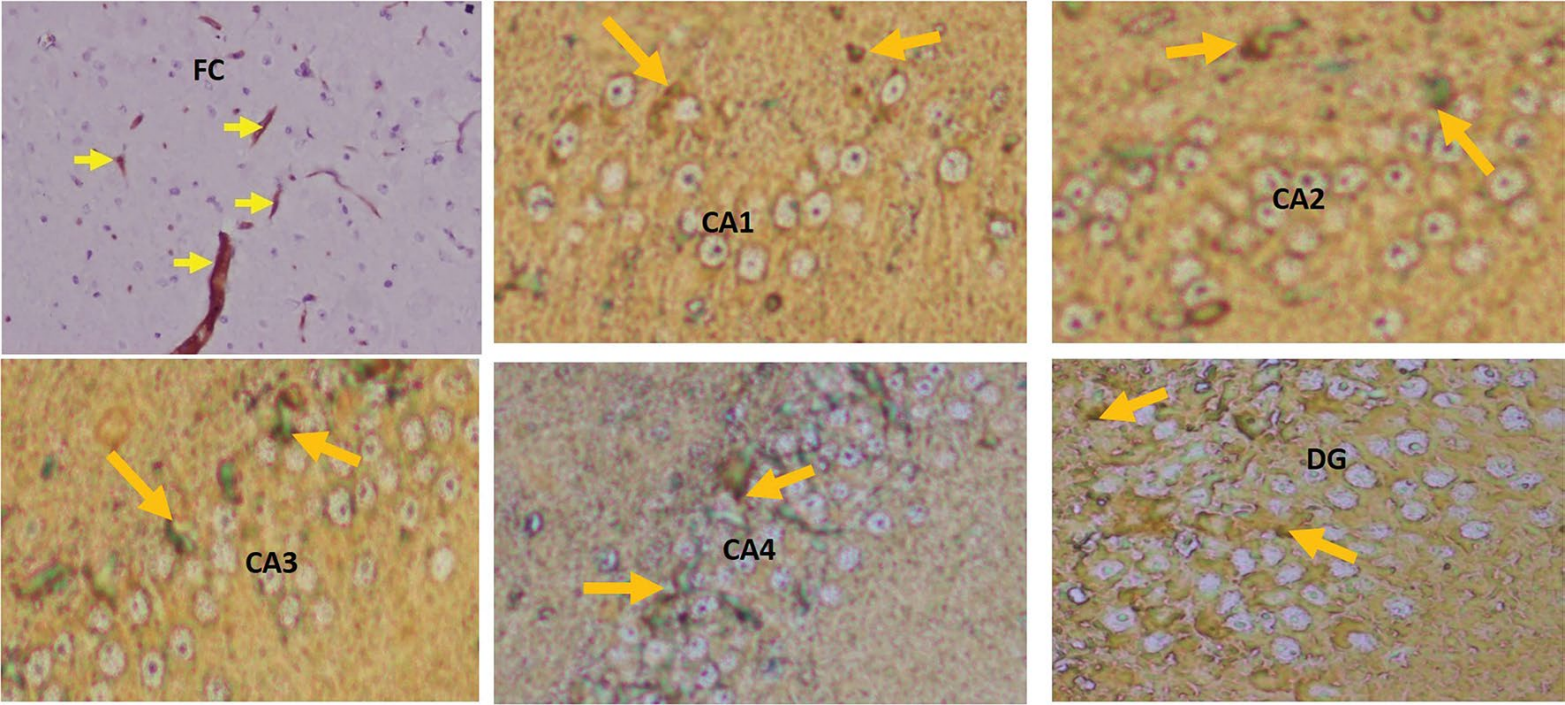
40 × view of the Aβ plaques (arrows) observed at the FC, CA1, CA2, CA3, CA4, and DG regions (mouse monoclonal anti Aβ antibody immunohistochemical staining; FC-frontal cortex; CA-cornu ammonis; DG-dentate gyrus)

**Fig. 10:**
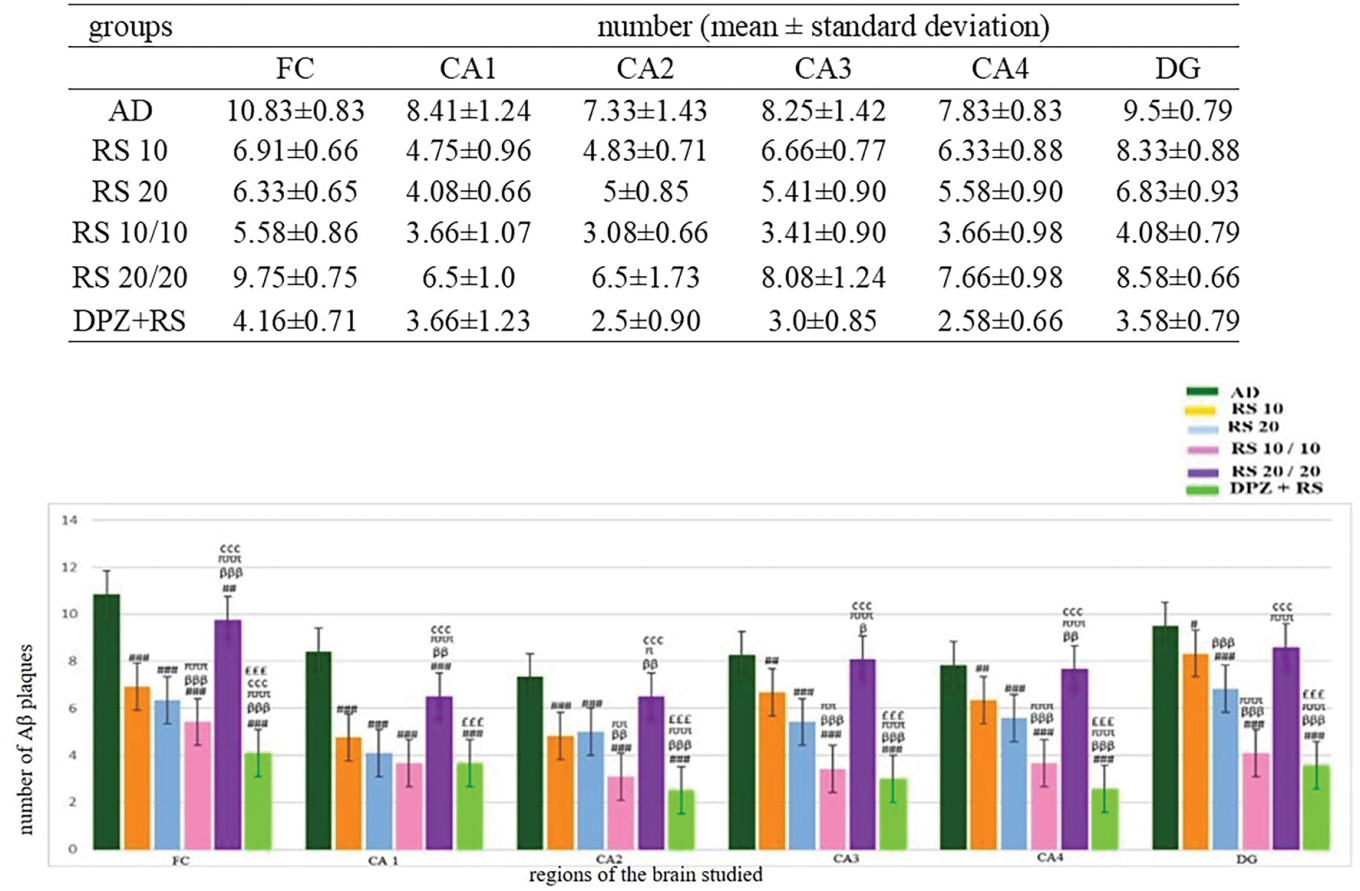
Comparsion of number of Aβ plaques among the groups (One-way ANOVA and post-hoc test, Bonferroni method, ^#^Vs AD, ^###^*p* < 0.001, ^##^*p* < 0.01, ^#^*p* < 0.05; ^β^Vs RS 10, ^βββ^*p* < 0.001, ^ββ^*p* < 0.01, ^β^*p* < 0.05; ^π^Vs RS 20, ^πππ^*p* < 0.001, ^ππ^*p* < 0.01, ^π^*p* < 0.05; ^¢^Vs RS 10/10, ^¢¢¢^*p* < 0.001, ^¢¢^*p* < 0.01, ^¢^*p* < 0.05; ^£^Vs RS 20/20, ^£££^*p* < 0.001, ^££^*p* < 0.01, ^£^*p* < 0.05)

The Aβ plaques observed at the CA regions of hippocampus, dentate gyrus and frontal region of cerebrum are given in Fig. 7. The Fig. 8 represents the same in various groups, which were studied in this research. It was observed that, Aβ plaques were observed to be diffuse in the AD model. In few locations, they were compactly arranged (Fig. 9). The number of plaques and statistical comparison of all the groups of this study showing the number of plaques at the frontal region and hippocampal formation is shown in Fig. 10. Table 1 shows the ‘*p*’ values of the groups compared for the number of Aβ plaques in this study.

**Table 1:** ‘*p*’ values of groups compared for the number of Aβ plaques in this study

Groups compared	FC	CA1	CA2	CA3	CA4	DG
AD Vs. RS 10	0.000000072	0.000000000037	0.000012	0.0062	0.0014	0.0127
AD Vs. RS 20	0.0000000000021	0.000000000000066	0.000048	0.00000009	0.0000005	0.0000000004
AD Vs. RS 10/10	0.0000000000000014	0.0000000000000014	0.0000000000022	0.0000000000000005	0.0000000000000002	0.0000000000000002
AD Vs. RS 20/20	0.487	0.00045	1	1	1	0.116
AD Vs. RS + DPZ	0.0000000000000002	0.0000000000000014	0.000000000000013	0.0000000000000002	0.0000000000000002	0.0000000000000002
RS 10 Vs. RS 20	0.19	1	1	0.681	0.621	0.00043
RS10 Vs. RS 10/10	0.00068	0.205	0.0045	0.0000000018	0.000000004	0.0000000000000002
RS10 Vs. RS 20/20	0.00035	0.0017	0.0082	0.0214	0.0067	1
RS10 Vs. RS + DPZ	0.000000034	0.205	0.000048	0.000000000031	0.000000000000023	0.0000000000000002
RS20 Vs. RS 10/10	1	1	0.0013	0.002	0.00002	0.00000000015
RS20 Vs. RS 20/20	0.000000016	0.0000055	0.025	0.00000047	0.000003	0.000027
RS20 Vs. RS + DPZ	0.00068	1	0.000012	0.0000049	0.0000000001	0.00000000000032
RS10/10 Vs. RS 20/20	0.000000000009	0.00000011	0.0000000038	0.0000000000000024	0.0000000000000015	0.0000000000000002
RS10/10 Vs. RS + DPZ	0.19	1	1	1	0.0564	1
RS20/20 Vs. RS + DPZ	0.00000000000000035	0.0000001	0.00000000002	0.0000000000000002	0.0000000000000002	0.0000000000000002

One-way ANOVA and post-hoc test; significance

*p* < 0.05; *p* value adjustment: Bonferroni method

**Fig. 11:**
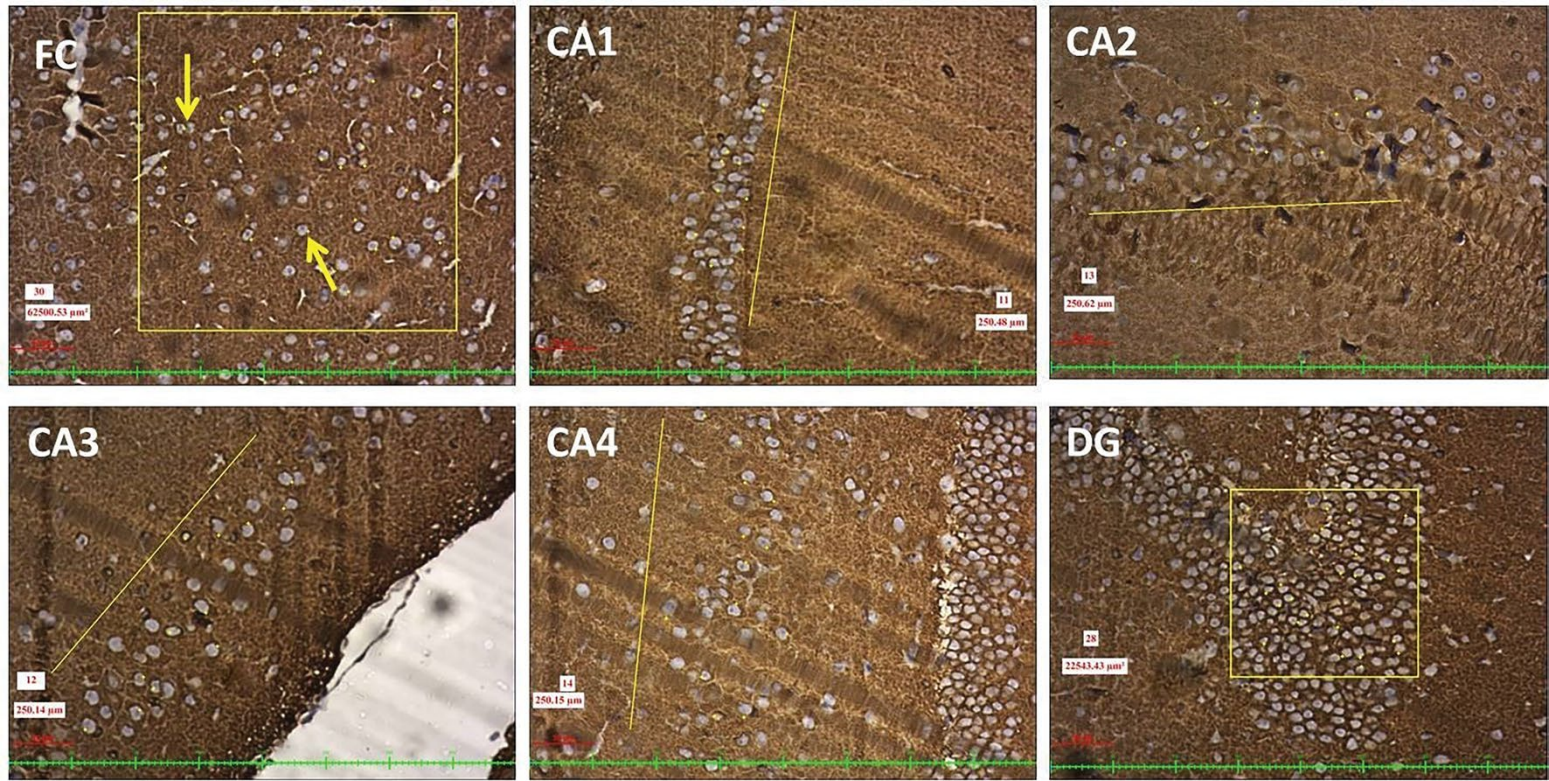
20 × view of the tau-positive neurons observed at the FC, CA1, CA2, CA3, CA4, and DG regions (polyclonal rabbit anti-tau antibody immunohistochemical staining; lines dictate the length of cornu ammonis region selected for the counting; squares are the area of the frontal cortex and dentage gyrus, selected for counting; the tau-positive neurons are shown as arrows)

**Fig. 12:**
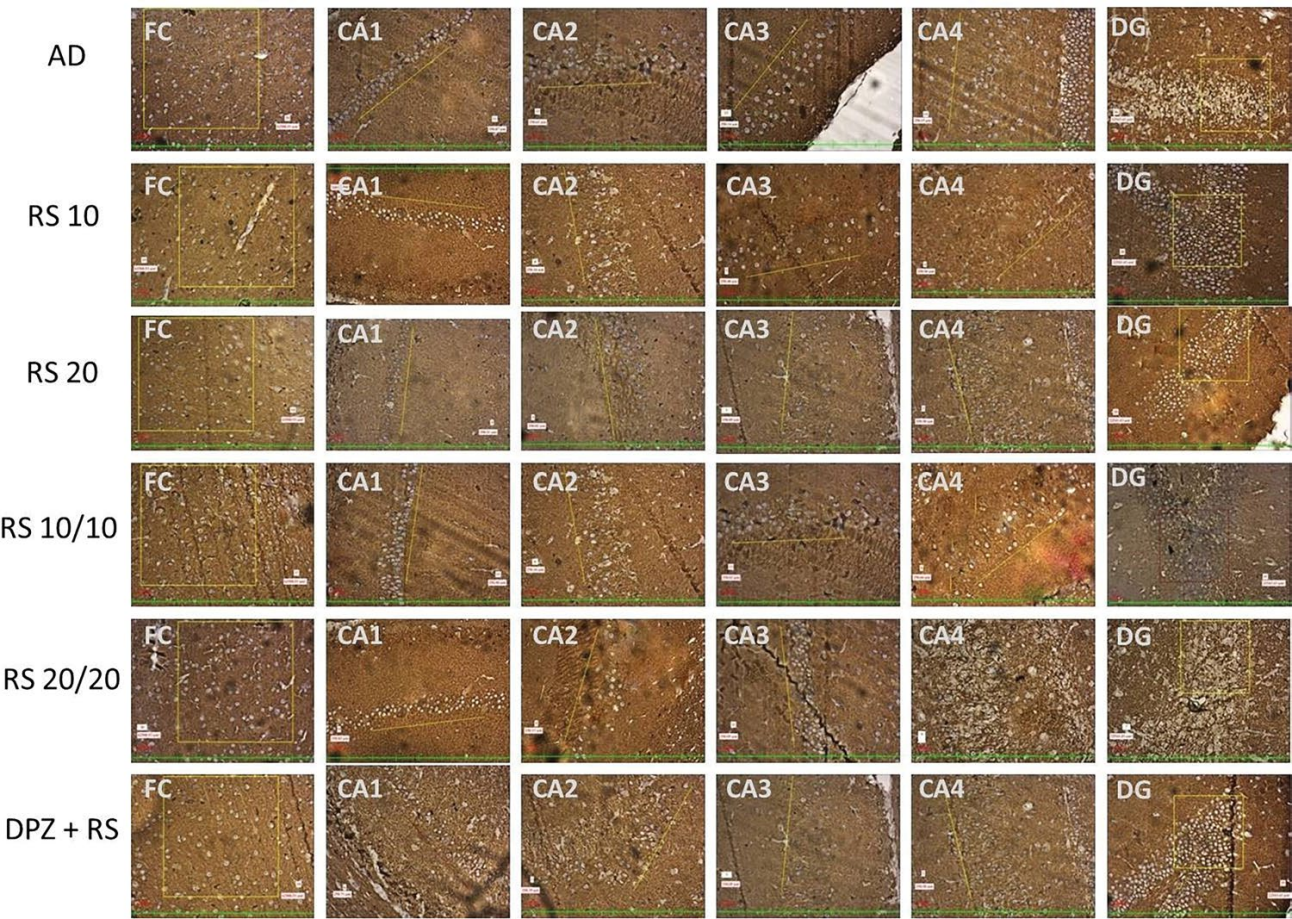
20 × view of the polyclonal rabbit anti-tau antibody immunohistochemical staining in different groups of this study showing the tau-positive neurons observed (FC-frontal cortex; CA-cornu ammonis; DG-dentate gyrus)

**Fig. 13:**
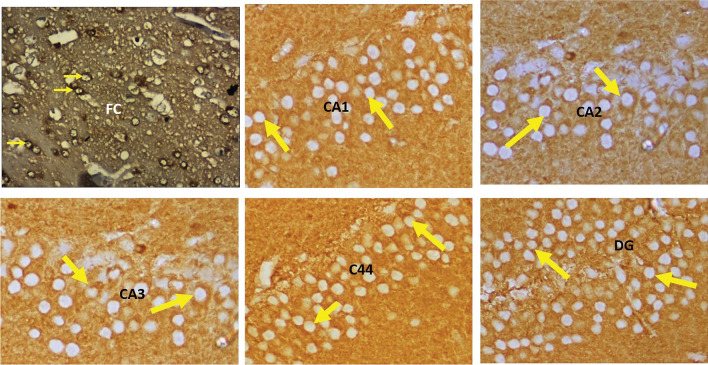
40 × view of the tau-positive neurons (arrows) observed at the FC, CA1, CA2, CA3, CA4 and DG regions (polyclonal rabbit anti-tau antibody immunohistochemical staining; FC-frontal cortex; CA-cornu ammonis; DG-dentate gyrus)

**Fig. 14:**
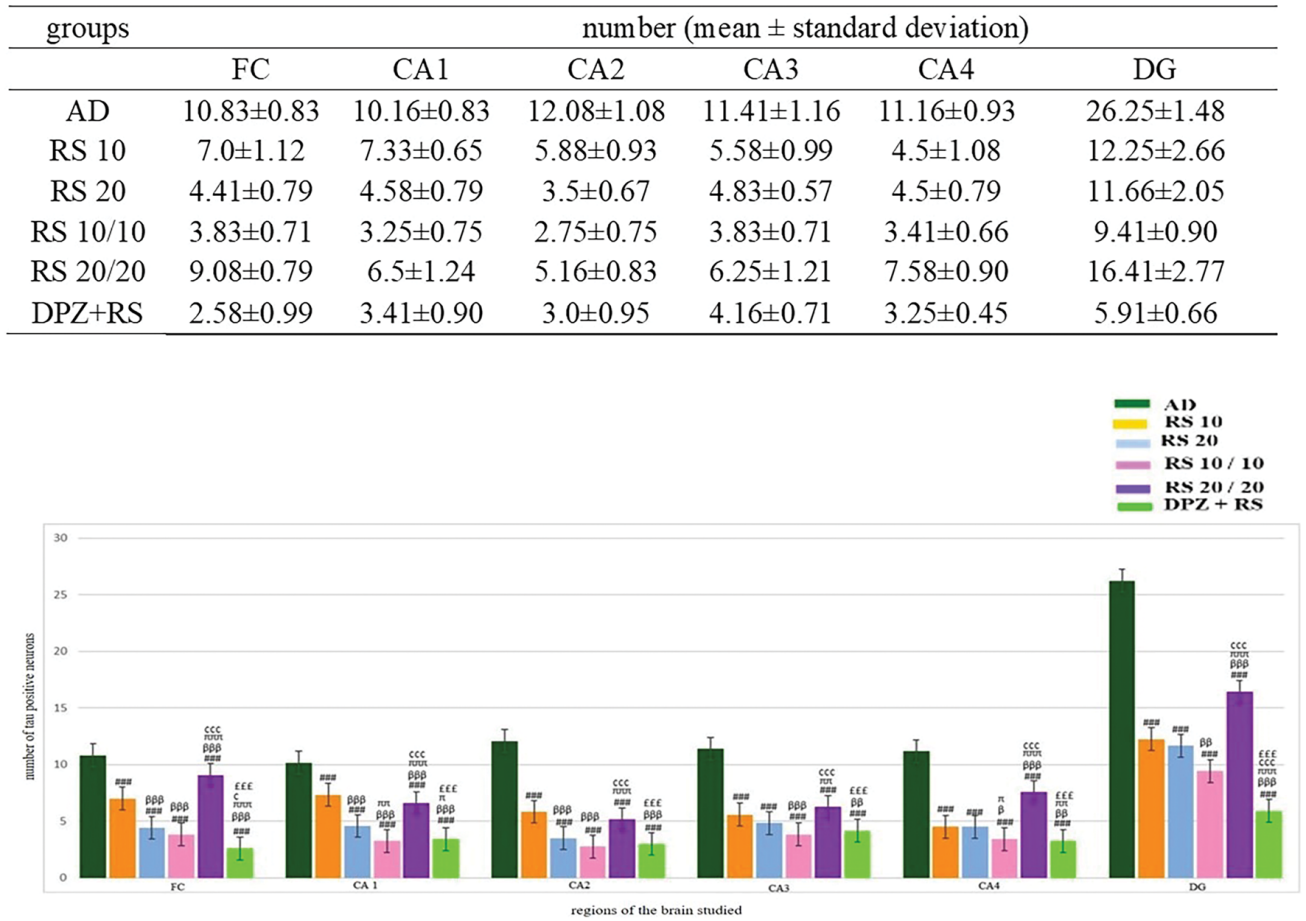
Comparison of number of tau-positive neurons among the groups (One-way ANOVA and post-hoc test, Bonferroni method, ^#^Vs AD, ^###^*p* < 0.001, ^##^*p* < 0.01, ^#^*p* < 0.05; ^β^Vs RS 10, ^βββ^*p* < 0.001, ^ββ^*p* < 0.01, ^β^*p* < 0.05; ^π^Vs RS 20, ^πππ^*p* < 0.001, ^ππ^*p* < 0.01, ^π^*p* < 0.05; ^¢^Vs RS 10/10, ^¢¢¢^*p* < 0.001, ^¢¢^*p* < 0.01, ^¢^*p* < 0.05; ^£^Vs RS 20/20, ^£££^*p* < 0.001, ^££^*p* < 0.01, ^£^*p* < 0.05)

Tau-positive neurons observed at the frontal region and hippocampal formation in this study are given in Fig. 11. The same at the different groups of this study are given in Figs. 12, 13 shows the 40X vision of the tau-positive neurons at the frontal lobe and hippocampal formation. The number of tau-positive neurons and their comparison among all the groups of this study are shown in Fig. 14. Table 2 shows the ‘*p*’ values of groups compared for the number of tau-positive neurons in this study.

**Table 2:** ‘*p*’ values of groups compared for the number of tau-positive neurons in this study

Groups compared	FC	CA1	CA2	CA3	CA4	DG
AD Vs. RS 10	0.000000000000012	0.0000000006	0.0000000000000002	0.0000000000000002	0.0000000000000002	0.0000000000000002
AD Vs. RS 20	0.0000000000000002	0.0000000000000002	0.0000000000000002	0.0000000000000002	0.0000000000000002	0.0000000000000002
AD Vs. RS 10/10	0.0000000000000002	0.0000000000000002	0.0000000000000002	0.0000000000000002	0.0000000000000002	0.0000000000000002
AD Vs. RS 20/20	0.00013	0.0000000000001	0.0000000000000002	0.0000000000000002	0.00000000000001	0.0000000000000002
AD Vs. RS + DPZ	0.0000000000000002	0.0000000000000002	0.0000000000000002	0.0000000000000002	0.0000000000000002	0.0000000000000002
RS 10 Vs. RS 20	0.000000015	0.000000001	0.0000002	0.786	1	1
RS10 Vs. RS 10/10	0.00000000002	0.0000000000000005	0.00000000004	0.00029	0.0329	0.0097
RS10 Vs. RS 20/20	0.0000038	0.6168	1	1	0.000000000004	0.000025
RS10 Vs. RS + DPZ	0.0000000000000002	0.0000000000000035	0.0000000007	0.0059	0.0071	0.00000000039
RS20 Vs. RS 10/10	1	0.0066	0.6215	0.157	0.0329	0.088
RS20 Vs. RS 20/20	0.0000000000000002	0.000008	0.00027	0.0059	0.000000000004	0.0000014
RS20 Vs. RS + DPZ	0.000055	0.0281	1	1	0.0071	0.0000000081
RS10/10 Vs. RS 20/20	0.0000000000000002	0.0000000000023	0.00000008	0.00000032	0.0000000000000002	0.000000000012
RS10/10 Vs. RS + DPZ	0.0148	1	1	1	1	0.00056
RS20/20 Vs. RS + DPZ	0.0000000000000002	0.000000000015	0.000001	0.00001	0.0000000000000002	0.0000000000000002

One-way ANOVA and post-hoc test; significance

*p* < 0.05; *p* value adjustment: Bonferroni method

### Estimation of Aβ plaques at the frontal cortex (Fig. 10, Table 1)

In all treatment groups, Aβ plaques were decreased in comparison to the group AD (*p* < 0.001). RS + DPZ groups revealed reduction in the number of Aβ plaques when compared to all other groups significantly (*p* < 0.001) showing the maximum efficacy. It was interesting to observe that, 20 mg prophylactic group exhibited higher number of plaques in contrast to 10 mg prophylaxis dose (*p* < 0.001).

### Estimation of Aβ plaques at CA1 region (Fig. 10, Table 1)

The Aβ plaques were decreased in contrast to the AD-induced group in all the groups except the 20 mg RS prophylactic group (*p* < 0.001). Here RS 10 mg prophylactic group and RS + DPZ combination groups have shown equal effect. Though the number of plaques is lesser in these groups than other groups (groups 1,2,3), there was no statistical significance (*p* > 0.05).

### Estimation of Aβ plaques at the CA2 region (Fig. 10, Table 1)

The Aβ plaques were less in number than the AD group in all other groups. However, the decrease in the number was not significant in 20 mg RS prophylactic group. Here also RS + DPZ combination groups have shown maximum efficacy than the other groups (*p* < 0.001).

### Estimation of Aβ plaques at CA3 region (Fig. 10, Table 1)

The reduction in number of Aβ plaques were observed in all the groups, compared to the AD group. But the decrease was not statistically significant in 20 mg RS prophylactic group. Here also RS + DPZ combination groups have shown maximum efficacy than the other groups (*p* < 0.001), however the difference between the RS 10 mg prophylactic and RS + DPZ group was not significant statistically.

### Estimation of Aβ plaques in CA4 region (Fig. 10, Table 1)

In all treatment groups, amyloid plaques were decreased than the AD group except 20 mg prophylactic group. The decrease in the number was not staiststically significant in RS 10 mg group, compared to AD group (*p* > 0.05).

### Estimation of Aβ plaques in dentate gyrus (Fig. 10, Table 1)

In all treatment groups, it was observed that, except 20 mg prophylactic group Aβ plaques were decreased in all the groups than the AD group. And in 20 mg, 10 mg prophylaxis and RS + DPZ groups the decrese in the number was highly significant (*p* < 0.001).

### Estimation of tau-positive neurons (NFT) in frontal cortex (Fig. 14, Table 2)

All the treatment groups showed reduction in the quantity of tau-positive neurons in comparison to AD model (*p* < 0.001). Further, tau-positive neurons were less in groups 20 mg, 10 mg prophylaxis and RS + DPZ than the 10 mg (*p* < 0.001). However, they were higher in the 20 mg prophylactic subgroup (*p* < 0.001). Combination of RS and DPZ showed significantly lesser number amongst all the groups.

### Estimation of tau-positive neurons (NFT) in CA1 region (Fig. 14, Table 2)

The tau-positive neurons significantly decreased in all the treated groups in comparison to the AD model (*p* < 0.001). Here, 20 mg RS prophylaxis indicated lower efficacy by showing more numbers of tau-positive neurons when compared to other groups (*p* < 0.001). RS 10 mg prophylactic dose and RS + DPZ groups showed equal potential.

### Estimation of tau-positive neurons (NFT) in CA2 region (Fig. 14, Table 2)

Compared to the AD group, all groups treated with RS showed a decrease in the tau-positive neurons (*p* < 0.001). However, comparison between the group 10 mg treated and 20 mg prophalyxis and also the comparison between the 10 mg prophylaxis and RS + DPZ could not show statistical significance (*p* > 0.05).

### Estimation of tau-positive neurons (NFT) in CA3 region (Fig. 14, Table 2)

The number of tau-positive neurons were decreased significantly in all the treatment groups (*p* < 0.001). Similar to CA1 region, the numbers were same in group 4 and 6. And the 20 mg prophylactic group more tau-positive neurons than other treatment groups.

### Estimation of tau-positive neurons (NFT) in CA4 region (Fig. 14, Table 2)

Though the tau-positive neurons were dcreased in all the treatment groups, the decrease in the number in 20 mg prophylaxis was less significant (*p* < 0.01). The numbers were minimum in RS 10 mg prophylactic group. However, the difference between the 10 mg prophylactic group and the RS + DPZ group was not statistically significant (*p* > 0.05).

### Estimation of tau-positive neurons (NFT) in dentate gyrus (Fig. 14, Table 2)

The tau-positive neurons were found less all the groups when compared to AD group (*p* < 0.001). Amongst these treated groups, the number was more in RS 20 mg prophylactic group (*p* < 0.001). Similar to other region observation, the number was minimal in RS + DPZ combination.

## Discussion

Though senile plaques are observed in the aged brain, the presence of an excessive number of such plaques is a diagnostic feature of AD (Walker 2020; Nelson et al. 2012). Masters and Beyreuther (2006) made observations that protein was the main content of these plaques. Initially, these proteins were named as the β protein, A4 or β/A4, which were termed as Aβ in the later days. Recently the Aβ proteins are popularly named Aβ plaques (Benson et al. 2018). Further, the accumulated plaques will also induce the oxidative damage to the nervous tissue. Along with the extracellular Aβ plaque deposition, another pathophysiology involved in the AD is intraneuronal accumulation of hyperphosphorylated tau protein as NFT, which might have also resulted from the oxidative stress (Huang et al. 2016). In our study, quantitative estimation of the β-amyloid plaques and tau-positive neurons was done to assess the role of these in the AD. RS is a potent antioxidant, which is chosen as a drug in this study against the colchicine-induced AD model to evaluate its efficacy in minimizing or preventing the β-amyloid plaque accumulation and formation of NFTs. In the human clinical trials, it was observed that RS dampens the accumulation of Aβ and decreases the neuroinflammation (Gu et al. 2021). However, Jang et al. (2021) observed elevation in Aβ after the administration of middle doses of RS in an AD cell model. Chen et al. (2019) demonstrated that, RS can decrease the amyloid plaque deposition in Tg6799 mice. They demonstrated that RS administration has significantly decreased the amyloid plaques, when estimated with the thioflavin S staining. Ge et al. (2012) documented that, RS in different states can directly blend with Aβ and their study offered new insight in the neuroprotective role of RS. In our study, it was observed that RS decreased the formation of plaques at the hippocampal regions and the frontal cortex (*p* < 0.001). Thus, our findings with RS administration are in line with the reports published by the above authors.

Hyperphosphorylation is the major hallmark of AD. RS, when given in low doses for the short term, can prevent hyper-phosphorylation in SAMP8 mice by inhibiting the tau kinases like, CDK5, CDK3B. But when RS was given for long term, it could prevent phosphorylation, however, inhibition of intermediate signals was lost, because of the long time use of the treatment (Porquet et al. 2013). RS decreased the hyperphosphorylated tau in brain sections, but promoted the tau accumulation in more soluble form (Porquet et al. 2013). This explains that RS can bind to hyperphosphorylated tau in a more stable form preventing the tangle formation. Means et al. (2020) reported that RS protected the death of astrocytes in the optic nerve from oxidative stress by tau dephosphorylation. Schweiger et al. (2017) opined that RS promotes tau dephosphorylation by the interference of MID1-PP2A complex. Nalagoni et al. (2016) observed that RS decreased the enlarged cells, NFT’s and vacuolar spaces in the cortical neurons, which were induced by the administration of aluminum and fluoride in rats.

The ROS are involved in exacerbation of the inflammation and antioxidants are capable of interfering with the production and inhibition of these ROS (Saraf et al. 2022). Presence of antioxidants will help the neurons against the oxidative damage and Aβ accumulation (Ma et al. 2014). Joy et al. (2019) used n-acetyl cysteine as an antioxidant and could observe that, this decreased the accumulation of tau in the neurons. However, they also observed that, there was no decrease in the Aβ accumulation. In our study, prophylactic dose of RS has shown the efficacy of prevention of plaque formation, proving that presence of antioxidants will prevent ROS production. However, in our study, when lower dose of RS was given as a prophylactic agent, there was less formation of plaques. Supporting our finding, Porquet et al. (2013) reported that RS, when given with lower doses, it can reduce the amyloid deposition, by favouring the non-amyloidogenic pathway in SAMP8 mice. RS being a potential antioxidant as well as an inducer of antioxidant enzymes in the brain tissue, could be a promising treatment drug as well as a prophylactic agent in AD. Our study has shown that the oxidative stress caused by the colchicine will lead to the NFT formation. Treatment with RS has shown less formation of NFT and a lower dose of prophylactic dose of RS could prevent the tangle formation. DPZ is beneficial and in use globally for the cognitive impairment and the clinical status has been improved in patients diagnosed with dementia including the Lewy body dementia (Mori et al. 2024). The present study also observed that RS and DPZ combination have more efficacies in reducing the plaque formation and tau-positive neurons (*p* < 0.001).

The present study observed that, oxidative stress caused the intra cellular accumulation of NFT’s and extracellular accumulation of Aβ plaques. These were decreased by the RS due to the remodeling and conversion of oligomers and fibrillary Aβ into non-toxic form. In the present study, we used different doses of RS including the prophylactic doses. These were considered as novel as the previous reports of different doses of RS are not available in the scientific literature. So, the comparison of different doses of RS could not be possible with the previous publications by fellow researchers. In this study, it was observed that, when RS was used as the prophylaxis, the lower dose was more beneficial than the higher dose. Hence lower dose of RS can be used effectively against plaque formation. RS may behave like a pro-oxidizing agent and becomes a cytotoxic molecule at higher doses (Martins et al. 2014; De la Lastra and Villegas 2007). Probably, this may be the reason for the non-beneficial effects with the longer duration of high dose of RS, which is observed in our study. The oxidative damage level and antioxidant activity of RS were already reported in our previous study (Rao et al. 2021) and the pro-oxidant effect of the higher dose of RS was considered, as this higher dose was not found beneficial.

The combined treatment of RS and DPZ is also a novelty in this study, as the reports are not available with these two drugs together. DPZ, rivastigmine, and galantamine are the three AChE inhibitors, which were approved by the US FDA to manage the symptoms of AD. However, these drugs did not prevent the disease progression. There are few side effects like weakness, muscular cramps, and urinary incontinence, which are being reported with the administration of these drugs, which limits their usage in the advanced AD. Hence, a novel molecule that can prevent the formation of Aβ and act as an antioxidant to ameliorate AD progression is of paramount importance (Tripathi et al. 2019b). Since AD is a multifactorial disease, multidrug regimen would be more effective in preventing and treating the AD. Since RS is showing dose dependent action, proper formulation and dosage is essential. Though RS is widely used as an antioxidant supplement for prophylaxis, the present study has proved its therapeutic effect as well by decreasing the Aβ and NFTs. Hence RS with the combination of DPZ could be a promising therapy in ameliorating the symptoms of AD and the specialists treating the AD patients may consider this regime.

However, this study has certain limitations like, this was actually an AD mimicking model, which was accomplished by the intraventricular injection of colchicine. Hence, the results of this study may not be generalized to human AD patients. The accurate AD model can be obtained with a transgenic model. Analysis of total antioxidant levels in the brain homogenate would give further information about the effect of RS on intrinsic antioxidants. For labeling and better estimation of β amyloid plaques, more sensitive stains like Gallyas silver staining, Thioflavin-S staining or Western blot methods can be used. The future implications of our present research can also include monitoring the proper dosage of RS or to modify the chemical structure of RS.

## Conclusion

The present study observed that RS 10 mg, 20 mg treatments and prophylactic dose of 10 mg have decreased the deposition of NFTs and Aβ plaques, showing the neuroprotective effect of RS. However, higher dose of RS, when given as 20/20 mg, showed toxic effects and this may be due to its pro-oxidant effect. The combination therapy of RS and DPZ was more beneficial, which indicates the synergistic effect of these two drugs. The future direction of this research can include studying the effect of RS in transgenic animal model of AD. Since RS is found to be non-beneficial with a higher dose, administered for a long duration, its pro-oxidant effect could be analysed. As our study observed the dose dependent effect of RS, it can be better further analyzed with the clinical trials. The combination of RS and DPZ could also be implemented in the human clinical trials, which can fill the existing treatment gap in the therapy of AD.
